# Supplementary material for: MetaRibo-Seq measures translation in microbiomes
Source: Nat Commun. 2020 Jun 29;11:3268. doi: 10.1038/s41467-020-17081-z (PMC7324362; doi:10.1038/s41467-020-17081-z)
Supplement: Supplementary file 10 — Supplementary Data 7 [file 41467_2020_17081_MOESM10_ESM.zip › File2/Confidence_High_Taxonomy/157156_out.krona.html]

Javascript must be enabled to view this page.

members
magnitude
magnitudeUnassigned
count
unassigned
taxon
rank

157156\_out

45

2
45
superkingdom

phylum
45
976

45
200643
class

171549
45
order

family
171552
45

838
1
genus

species

SRS014271\_contig\_number\_21020
1
425941

44
1283313
genus

76122

SRS013663\_contig\_number\_contig-100\_1102.167424SRS013836\_contig\_number\_contig-100\_884.185445SRS013950\_contig\_number\_18814SRS014107\_contig\_number\_1027SRS014691\_contig\_number\_22852SRS015686\_contig\_number\_7547SRS015762\_contig\_number\_25910SRS017227\_contig\_number\_61202SRS017634\_contig\_number\_contig-100\_331.159978SRS018157\_contig\_number\_50485SRS018479\_contig\_number\_55865SRS018739\_contig\_number\_24084SRS018826\_contig\_number\_28126SRS019029\_contig\_number\_25658SRS019488\_contig\_number\_1720SRS019696\_contig\_number\_14359SRS022143\_contig\_number\_8526SRS024318\_contig\_number\_27850SRS024441\_contig\_number\_23605SRS045197\_contig\_number\_41577SRS047634\_contig\_number\_47627SRS047824\_contig\_number\_29362SRS050244\_contig\_number\_25805SRS057205\_contig\_number\_19989SRS062544\_contig\_number\_26329SRS063215\_contig\_number\_13731SRS064014\_contig\_number\_31317SRS075947\_contig\_number\_18392SRS077022\_contig\_number\_30587SRS077216\_contig\_number\_19222SRS077520\_contig\_number\_70258SRS101382\_contig\_number\_22088SRS101445\_contig\_number\_48579SRS103971\_contig\_number\_38679SRS104842\_contig\_number\_59488SRS1055101\_contig\_number\_17590SRS143529\_contig\_number\_18275SRS144512\_contig\_number\_28814SRS144609\_contig\_number\_31073SRS144621\_contig\_number\_32313SRS147386\_contig\_number\_35844SRS148205\_contig\_number\_24682SRS893227\_contig\_number\_4227SRS893232\_contig\_number\_contig-100\_150.175662
44
species
